# Indoxyl Sulfate, a Uremic Endotheliotoxin

**DOI:** 10.3390/toxins12040229

**Published:** 2020-04-05

**Authors:** Guillaume Lano, Stéphane Burtey, Marion Sallée

**Affiliations:** 1 C2VN, Aix Marseille Univ, INSERM, INRAE, 13005 Marseille, France; guillaume.lano@ap-hm.fr (G.L.); marion.sallee@ap-hm.fr (M.S.); 2 Centre de Néphrologie et Transplantation Rénale, AP-HM, Hôpital de la Conception, 147 Bd Baille, 13005 Marseille, France

**Keywords:** chronic kidney disease, indoxyl sulfate, cardiovascular disease, endothelial dysfunction, aryl hydrocarbon receptor

## Abstract

Chronic kidney disease (CKD) is associated with a high prevalence of cardiovascular diseases. During CKD, the uremic toxin indoxyl sulfate (IS)—derived from tryptophan metabolism—accumulates. IS is involved in the pathophysiology of cardiovascular complications. IS can be described as an endotheliotoxin: IS induces endothelial dysfunction implicated in cardiovascular morbidity and mortality during CKD. In this review, we describe clinical and experimental evidence for IS endothelial toxicity and focus on the various molecular pathways implicated. In patients with CKD, plasma concentrations of IS correlate with cardiovascular events and mortality, with vascular calcification and atherosclerotic markers. Moreover, IS induces a prothrombotic state and impaired neovascularization. IS reduction by AST-120 reverse these abnormalities. In vitro, IS induces endothelial aryl hydrocarbon receptor (AhR) activation and proinflammatory transcription factors as NF-κB or AP-1. IS has a prooxidant effect with reduction of nitric oxide (NO) bioavailability. Finally, IS alters endothelial cell and endothelial progenitor cell migration, regeneration and control vascular smooth muscle cells proliferation. Reducing IS endothelial toxicity appears to be necessary to improve cardiovascular health in CKD patients.

## 1. Background

**Figure 1:**
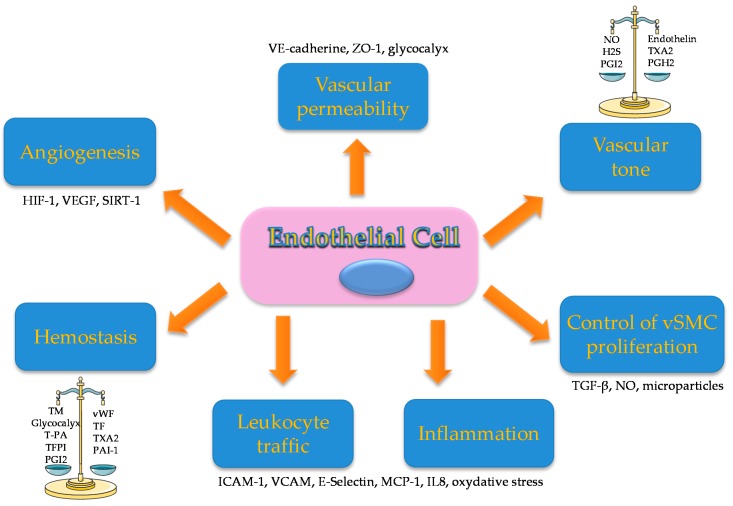
Endothelium functions: The endothelium has many different functions in the regulation of major processes: Hemostasis maintaining an equilibrium between pro and anticoagulant factors. Procoagulant factors: vWF (Von Willebrand factor), TF (tissue factor), Thromboxane A2 (TXA2), plasminogen activator inhibitor1 (PAI-1). Anticoagulant factors: Thrombomodulin (TM), glycocalyx, tissue plasminogen activator (T-PA), tissue factor plasminogen inhibitor (TFPI) and prostaglandin I2 (PGI2). Angiogenesis hypoxia inducible factor (HIF-1), vascular endothelium growth factor (VEGF) and Sirtuin 1 (SIRT-1). Vascular permeability with expression of junction proteins like VE-cadherin, zona occludens 1 (ZO-1) and glycocalyx. Vascular tone through production of vasodilator factors: nitric oxide (NO), hydrogen sulfide (H2S) and prostaglandin I2 (PGI2); vasoconstrictor factors: endothelin, TXA2 and prostaglandin H2 (PGH2). Vascular smooth muscle cells (vSMC) proliferation by secretion of: Tumor Growth Factorβ (TGF-β), NO and microparticles. Inflammation and leukocyte traffic by the production of intercellular adhesion molecule 1 (ICAM-1), vascular cell adhesion protein 1 (VCAM-1), E-selectin, membrane cofactor protein 1 (MCP-1), interleukin 8 (IL-8) and oxidative stress.

Endothelium was historically described as a “cellophane type” barrier that separated blood from other organs and tissues. Despite the selective permeable interface separating vascular and interstitial compartment, several functions were described over the past 40 years. The endothelium is now a dramatic regulator of hemostasis, inflammation and vascular tone in collaboration with vascular smooth muscle cells (vSMC), via a nitric oxide (NO)-dependent pathway [1,2]. The endothelium also plays an important role in controlling leukocyte trafficking, metabolism, vascular permeability and angiogenesis (Figure 1).

We could estimate the number of endothelial cells to 6 10^11^ cells (2.5% of total body cells) [3] covering an area of 740 m^2^. Endothelial cells cover the whole cardiovascular system surface. Endothelial cell characteristics are different, depending on the type of vessel (microvessel vs macrovessels) or organ. Endothelial cells insure an antithrombotic and anti-inflammatory environment to the vessels and provide oxygen and nutrients to all tissues. NO, released by the endothelium, is a major regulator of vascular tone exerting a vasodilatory effect. NO plays a crucial role in protecting the endothelium as well as inhibiting proliferation and migration of vSMC, expression of adhesion molecules and platelet aggregation [4]. Endothelial dysfunction is historically described by Furchgott [5] as the defect of NO production by the endothelium. Alteration in endothelial function inducts morphologic atherosclerotic changes and promotes cardiovascular complications.

## 2. Endothelial Activation is Associated with Impaired Vascular Function and Cardiovascular Disease

Endothelial cells are exposed to several external stresses resulting in endothelial activation. This activation induces “an unhealthy endothelium”, commonly called endothelial dysfunction (ED). Endothelial dysfunction plays important role in various acute and chronic diseases. Unhealthy endothelium is defined by the loss of physiological functions of endothelial cells leading to the loss of vascular homeostasis [4,6].

In response to an injury, the endothelium is activated resulting in a pro-inflammatory and procoagulant state with an abnormal vascular tone. In this state, the endothelium expresses adhesion molecules and increases reactive oxygen species (ROS) production. If the aggressive process persists, the endothelium can suffer direct damage and lose its integrity. Circulating endothelial cells (CEC) detectable in the bloodstream originate from endothelial cell senescence and detachment [7]. In presence of vascular injury, endothelial progenitor cells are recruited from the bone marrow or adjacent endothelial cells. Endothelial dysfunction impairs the endothelial progenitor cell mobilization [8,9]. One of circulating markers of EC injury is endothelial microparticles. These particles are derived from activated cells, apoptotic cells and whole endothelial cells. Endothelial dysfunction impairs endothelial cell-vSMC interactions and disrupts vSMC maturation, differentiation and proliferation.

Unhealthy endothelium is induced by endogenous factors (catecholamines, bradykinin, acetylcholine, histamine, thrombin, VEGF, cytokines (TNF-α or IL-1)), mechanical stimuli (turbulent shear stress) [10], bacterial lipopolysaccharides (LPS) and all external and environmental factors that increase ROS (free fatty acid oxidized LDL, hyperglycemia and advanced glycation end products (AGEs) [6,11], tobacco [12] and pollutants like dioxin [13]).

Endothelial dysfunction is associated with cardiovascular diseases promoting atherosclerosis and acute thrombosis [8] and endothelial cell-vSMC disruption are involved in the formation of atheromatous disorders [14]. Therefore, endothelial dysfunction plays a major role in numerous cardiovascular diseases as hypertension, coronary heart disease, heart failure, peripheral arterial disease, stroke and diabetes [6,15,16].

## 3. Chronic Kidney Disease is Associated with Cardiovascular Disease

Chronic kidney disease (CKD) is a common disease, with a prevalence of around 11% in high-income countries representing a major burden on the public health system [17]. Patients with CKD are at high risk of cardiovascular disease and this risk increases with kidney failure progression [18,19,20]. CKD is associated with an increase in thrombotic events (myocardial infarction, stroke, venous thrombo-embolism) and accelerates atherosclerosis which promotes ischemic heart disease, heart failure and peripheral vascular disease [21,22,23]. CKD is known to be associated with endothelial dysfunction. Endothelial dysfunction is described early during CKD. Clinical and biologic evidence of endothelial dysfunction in CKD is widely reported [24,25,26]. Flow mediated vasodilatation (FMD), a functional test of endothelial dysfunction, is impaired in patients with CKD [27]. Furthermore, endothelial microparticles, arterial stiffness and higher pulse wave velocity are increased in patients with CKD compared to those with normal kidney function [28]. Pulse wave velocity is described as an independent marker of cardiovascular mortality in hemodialysis patients [29]. Showing correlation of endothelial microparticles with impaired FMD and increased aortic pulse wave velocity [30,31]. In contrast, endothelial progenitor cell number is reduced [32,33] and contributes to impaired neovascularization [23,34] after injury.

Uremic indolic toxins of which indoxyl sulfate (IS) is the most extensively studied, are largely involved in the pathogenesis of cardiovascular diseases during CKD [35,36,37]. IS is derived from the tryptophan metabolism (indolic pathway). Tryptophan is digested by intestinal bacteria in indole. Subsequently, the absorbed indole is metabolized to IS in the liver [38]. Finally, IS is normally excreted in urine. IS is a protein-bound toxin, a large proportion is bound to albumin, but plasma levels of free IS can be measured separately. IS accumulates in kidney failure and its plasma concentration can reach 100 fold higher than that observed in healthy subjects [39] as the protein bound toxin cannot be cleared by dialysis [40,41]. Serum IS levels are inversely correlated with estimated Glomerular Filtration Rate (eGFR) [42].

## 4. Indoxyl Sulfate, a Uremic Endotheliotoxin: Clinical Evidence

**Figure 2:**
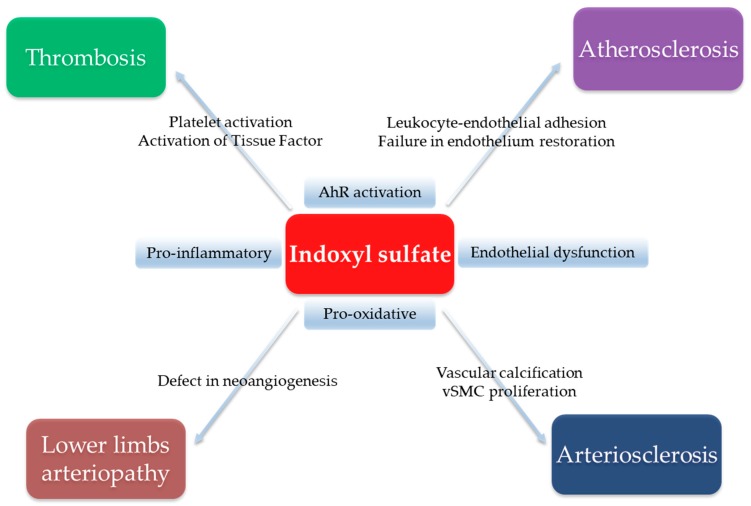
Indoxyl sulfate (IS) and cardiovascular pathophysiology. IS endothelial toxicity inducing cardiovascular disorders by multiple processes: pro-inflammatory, pro-oxidative, AhR activation and as a result endothelial dysfunction. Consequently, IS promotes thrombosis (through the activation of platelets and endothelial tissue factor); lower limb arteriopathy (arteriosclerosis and altered neoangiogenesis); atherosclerosis (increased adhesion of leukocytes and failure to restore the endothelium) and arteriosclerosis (via vascular calcification and vSMC proliferation induced by endothelial dysfunction).

Several studies have confirmed the link between plasma IS levels and cardiovascular morbidity and mortality in CKD patients (Figure 2).

First, Barreto et al. [43] demonstrated that IS concentration is a strong predictor of mortality and cardiovascular events in patients with different stages of CKD (from stage one to five days). More recently, Fan et al. [44] confirmed this association before the dialysis stage, independently of eGFR and nutritional status. Shafi et al. [45] studied this association in hemodialysis patients: in a post hoc analysis of the HEMO study, IS plasma levels were predictive of cardiac and sudden cardiac death in the subgroup of patients with lower albumin. A meta-analysis performed in 2015 by Lin et al. [46] found an association between free IS plasma levels and mortality but not with cardiovascular outcomes. This meta-analysis included five studies with heterogeneity in dialysis modalities (peritoneal dialysis or hemodialysis) and in the follow-up of patients. IS levels were correlated with an increased risk of arteriovenous fistula thrombosis after angioplasty in two studies [47,48]. These observational studies were limited by potential confounders between IS plasma levels and nutritional intake. In fact, low protein intake is associated with a poor prognosis in CKD [49]. On the other hand, reduced protein intake impacted negatively IS plasma levels [50,51]. Thus, low IS plasma levels may reflect a poor nutritional state rather than an improved uremic status. In patients with CKD, IS plasma levels are correlated with pulse wave velocity [43], vascular reactivity index [52], ankle brachial index, arteriosclerosis markers [53] and aortic calcification [43]. Moreover, the number of endothelial progenitor cells is inversely correlated with IS plasma levels [54,55]. This mechanism could impair neovascularization [48,49,50,51]. The endotheliotoxicity of IS was demonstrated in living kidney donors with an eGFR greater than 60 mL/mn/1.73 m^2^ and increased IS plasma levels. After two years of follow-up, the concentration of IS was correlated with FMD and an increase in intima media thickness [56]. These results confirm the role of IS as an endotheliotoxin before stage three CKD.

## 5. Indoxyl Sulfate, a Uremic Endotheliotoxin: Experimental Evidence

Studies evaluating the specific toxicity of IS in animals are sparse and are restricted to murine models. Furthermore, protocols and animal models are heterogeneous depending on the method of IS administration (intraperitoneally or orally), IS dose and models of kidney injury (normal function, subtotal nephrectomy, adenine diet) [57].

### 5.1. Evidence of a Role in Vascular Calcification

Chen et al. have shown that intraperitoneal administration of IS increases aortic calcification in subtotal nephrectomized rats [58]. Similarly, IS administered orally promotes aortic calcification in hypertensive rats with normal kidney function [59,60]. More recently, Opdebeeck et al. [61] showed that exposure to IS in a rat model with CKD induced by adenine diet increases aortic calcification. The amount of aortic calcification is associated with IS plasma concentration.

### 5.2. Evidence for a Prothrombotic State

Karbowska et al. described that IS exposure induces thrombosis in two non-CKD animal models: by direct electric stimulation of carotid artery in the rat model after chronic oral exposure to IS [62] or after one injection of IS [63] and by laser-induced endothelial injury [62] in the mouse model after one injection of IS. In the rat model, they described a higher incidence of thrombosis and a heavier thrombus after one injection of IS [63]. With the highest dosage, IS decreased the clotting time, increased the total area of the thrombus and the maximum firmness of the clot compared to control. Similar evidence of thrombus formation was reported after chronic oral exposure to IS in rat model [62] and in the mice models with higher IS doses (more than 30 mg/kg) [63]. Recently, in a model of cancer-associated thrombosis, increased levels of IS were observed and associated with more extensive thrombosis [64] extending the endotheliotoxic role of IS beyond uremia.

### 5.3. Evidence of an Impaired Neovascularization

In subtotal nephrectomized mice fed indole (leading to an increase in circulating IS), reduced blood flow was observed on reperfusion following unilateral hindlimb ischemia as well as a lack of increase capillaries and small artery density compared to subtotal nephrectomized mice on a normal diet [65]. Furthermore, in this model, endothelial progenitor cell mobilization in muscle was reduced by CKD, especially after enrichment of indole diet [65]. Similarly, Wu et al. confirmed in mice that IS administration decreases the number of circulating endothelial progenitor cells [54].

## 6. Indoxyl Sulfate, a Uremic Endotheliotoxin: Interventional Studies

The orally administered spherical carbon adsorbent AST-120 reduces indole absorption through gastrointestinal sequestration. Thereby, AST-120 reduces IS plasma levels in patients with CKD [66]. AST-120 failed to improve survival in CKD patients [67] but until now, no study was designed to determine whether AST-120 improves cardiovascular outcomes [68]. A 24-month clinical study with AST-120 reduced the carotid intima media thickness and pulse wave velocity in non-dialysis patients with CKD [69]. Goto et al. [70] demonstrated that exposure to more than six month treatment with AST-120 was associated with a lower aortic calcification index in predialysis patients. Twenty four weeks of treatment with AST-120 improved ultrasound FMD and this was correlated with reduced IS plasma level [71]. In animal models, the impact of AST-120 has been well reported. In subtotal nephrectomized apoE-/-mice, AST-120 dramatically decreased aortic atherosclerosis after 16 weeks of treatment [72]. Eight weeks of AST-120 prevented the increase in pulse wave velocity in CKD mice [73]. Unfortunately, in these studies, the authors did not mention whether these effects were correlated with the reduction in IS blood concentrations [72,73]. In the hindlimb ischemia model in CKD mice, AST 120 prevented the reduced blood flow reperfusion and restored neovascularization in the ischemic limb [65].

## 7. Indoxyl Sulfate, a Uremic Endotheliotoxin: Molecular Mechanisms

**Figure 3:**
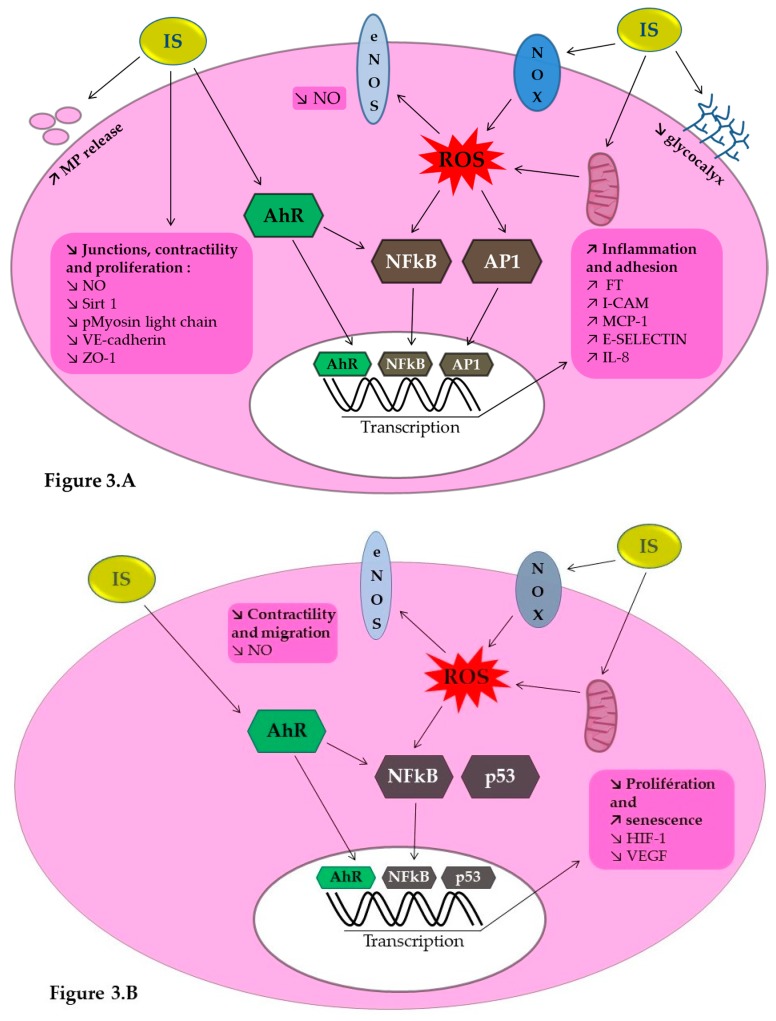
Molecular effects of indoxyl sulfate (IS) on endothelial cells (**3A**) and endothelial progenitor cells (**3B**). 3A: IS activates the aryl hydrocarbon Receptor (AhR) genomic pathway via AhR nuclear translocation and the AhR non genomic inflammatory pathway inducing Nuclear Factor κB (NFκB) and activating protein 1 (AP1) translocation. Activation of the NF-κB and AP-1 pathway is also promoted by reactive oxygen species (ROS) induced by IS. IS induces reactive oxygen species production by activation of the NADPH oxidase (NOX) in mitochondria and plasma membrane. All these pathways converge to increase the production of proteins involved in inflammation, thrombosis and leukocyte adhesion: tissue factor (TF), intercellular adherence protein (ICAM-1), membrane cofactor protein 1 (MCP1), E-selectin and interleukin 8 (Il-8). IS induces glycocalyx alteration and microparticle (MP) release, altering endothelial cell junctions, contractility and proliferation. This results in a decrease in nitric oxide (NO) production by endothelial NO synthase (e-NOS), and decreases Sirtuin 1 (SIRT-1), myosin light chain, VE-cadherin and zona occludens 1 (ZO-1). **3B.** IS promotes endothelial progenitor cell senescence decreasing proliferation through an altered production of hypoxia inducible factor 1 and vascular endothelial growth factor (VEGF) with a mechanism that involves the transcription factor p53.

Endothelial dysfunction is recognized as one of the first mechanisms leading to atherosclerosis and thrombosis. In this part, we will describe the main cellular pathological processes induced by IS which are involved in atherosclerosis, impaired neovascularization and thrombosis (Figure 3).

### 7.1. IS Implication in NO Production and Impaired Neovascularization

IS known to induce endothelial toxicity in vitro. An increase in endothelial microparticles [62], a decrease in NO bioavailability and an increase in reactive oxygen species (ROS) production [40,73,74,75,76] were reported when endothelial cell are exposed to IS. NO is a key mediator of endothelial cell function and angiogenesis. ROS production is induced by the activation of NADPH oxidase, a pro-oxidative enzyme [75,76] and by depletion of endothelial glutathione, known for its strong antioxidant effect [76]. IS is known to induce NADPH oxidase expression (NOX4) [75], and reduced production of NO by the endothelium limiting endothelial cell migration and tube formation [77,78,79]. In addition, IS inhibited the activation of the ERK MAP kinase pathway and myosin light chain involved in endothelial contractibility and migration [79]. In vivo, reducing the blood concentration of IS with AST 120 was associated with an improvement in NO bioavailability and better endothelial function [71,73].

IS plays a role in endothelial integrity. IS induces the expression of adhesion molecules via NF-κB pathway: E-selectin [80], intercellular adhesion molecule-1 (ICAM-1) and monocyte chemotactic protein-1 (MCP-1) [75]. Interestingly, the antioxidant N-acetylcysteine reverses these effects [75,80]. In patients with CKD, adhesion molecules such as soluble E-selectin are positively correlated to IS plasma levels [80]. IS also impacts the cell junction, decreasing endothelial VE-cadherin expression in HUVEC [71]. Arteries of CKD patients show a reduction in the cell junction proteins VE-cadherin and ZO-1 [81]. IS decreases endothelial progenitor cell proliferation, increases endothelial progenitor cell senescence, induces oxidative stress via a NO dependent pathway and alters endothelial progenitor cell mobilization and angiogenesis. Carmona et al. [82] described endothelial progenitor cell senescence induced by IS via p53 and NF-κB activation reducing the ability to form new vessels. In endothelial progenitor cells, IS decreases VEGF production in response to hypoxia [65]. Interestingly, treatment with AST-120 restores normal neovascularization [65].

### 7.2. IS Implication in Vascular Calcification

IS promotes VSMC calcification via IL-8 secretion by endothelial cells in the presence of inorganic phosphate. [83]. Endothelial microparticles released in presence of IS [84,85] induces VSMC proliferation and transforming growth factor β (TGFβ). This could play a role in intimal hyperplasia of vascular stenosis [84].

### 7.3. IS Implication in Thromboinflammation

Intravenous infusion of IS in non-CKD rats induces adhesion and extravasation of leukocytes similar to the stimulation of the innate immune system [86]. Moreover, a significant drop in blood flow was observed with IS perfusion related to an alteration in the endothelial glycocalyx [86]. A thinner endothelial glycocalyx increases leukocyte adhesion, induces vascular inflammation and the progression of atherosclerosis [87].

The clinical prothrombotic state observed in patients with CKD is identified by the increase in fibrinolysis markers such as d-dimers and thrombin-antithrombin complexes. This prothrombotic state is related to an increased level of procoagulant factors (factor VII, factor VIII, fibrinogen), factors implicated in fibrinolysis (plasminogen activator inhibitor type-1) or endothelial function (Von Willebrand Factor, thrombomodulin) [21]. The IS concentration is independently associated with endothelial prothrombotic factors (Von Willebrand Factor, thrombomodulin) and endothelial adhesion molecules (I-CAM and V-CAM) in a non-dialysis CKD cohort [26]. These circulating markers reflect endothelial activation by IS through production of ROS and activation of NF-κB [75].

The major marker of the procoagulant state in CKD patients is the activation of the endothelial tissue factor. Tissue factor is a transmembrane protein and the main initiator of thrombin formation [88]. The upregulation of tissue factor is associated with cardiovascular disorders [89]. In patients with CKD, IS plasma levels are correlated with circulating tissue factor and its procoagulant activity (determined by measuring the generation of factor Xa) [26,90,91]. Recently, Kolachalama et al. [48] showed a correlation between plasma tissue factor activity and arteriovenous fistula thrombosis in hemodialysis patients. In vitro, IS increases endothelial expression of tissue factor and its procoagulant activity secondary to activation of the aryl hydrocarbon receptor (AhR) [91]. We will describe this important pathway in the next section.

## 8. Indoxyl Sulfate, a Uremic Endotheliotoxin: Aryl Hydrocarbon Receptor Activation as a Key Mechanism

One of the recent major contributions in the understanding of cardiovascular complications in patients with CKD is the involvement of the aryl hydrocarbon receptor (AhR) pathway [91,92,93,94]. AhR is a transcription factor involved in biologic detoxification. When AhR is bound to a ligand, AhR translocates to the nucleus and modulates the expression of genes under the control of xenobiotic response elements, as part of the genomic pathway. After ligand binding to AhR this activates an inflammatory non genomic pathway leading to the activation of other transcriptions factors such as NF-κB and AP-1. Environmental pollutants and naturally occurring ligands such as tryptophan derivatives including IS are known to induce AhR activation [95]. The ability of serum from CKD patient to activate AhR in vitro is estimated through the AhR activating potential (AhR-AP) measurement which reflects the concentration of all AhR agonists in serum including IS. AhR-AP levels are inversely correlated to eGFR [93] and correlated with IS plasma levels and cardiovascular events [93]. Kolachalama el al [48] reported that arteriovenous fistula thrombosis in CKD patient is associated with higher serum AhR-AP. Furthermore, IS plasma concentration, serum AhR-AP and tissue factor activity were correlated in a cohort of 20 hemodialysis patients [90]. In a subtotal nephrectomy mouse model, serum AhR-AP is increased compared to control [93]. Moreover, IS administration in wild type mice induces AhR activation in the liver, kidney and aorta [93]. In vitro, IS at uremic concentrations enhances activation of AhR [91,93]. IS activates the AhR genomic pathway with AhR nuclear translocation [91] and the AhR inflammatory non genomic pathway [92]. In vitro inactivation or pharmacological inhibition of the AhR in endothelial cells reverses tissue factor induction by IS [90,91] and similarly in vivo in mice [64]. Other aspects of endothelial dysfunction induced by IS were related to AhR activation. Ito et al. [96] described a reduction of leukocyte recruitment induced by subcutaneous injection of IS in a endothelial specific AhR KO mouse model compared to wild type mice. In endothelial cells, the expression of E-selectin induced by IS is driven by an AhR-AP1 pathway [96]. Watanabe et al. [97] showed that MCP-1 expression induced by IS is reversed by AhR inhibition. Inactivation of the AhR pathway decreased ROS production by inhibiting NOX4 expression [97]. Moreover, Masai et al. [98] confirmed the implication of AhR in MCP-1 induction by IS via a ROS/MAPK/NFκB pathway. AhR inhibition prevent IS-induced HUVEC senescence via activation of NADPH and Sirt1 [99]. So, IS induced ED and a procoagulant state in HUVEC by AhR inflammatory pathways (via NF-κB and AP-1) and an increase in oxidant stress.

## 9. Conclusions

The uremic toxin indoxyl sulfate is an endotheliotoxin largely involved in the genesis of the cardiovascular disorders in patients with CKD. In endothelial cells, IS promotes pro-oxidant, pro-inflammatory and prothrombotic processes involved in endothelial dysfunction. This dysfunction induces arteriosclerosis, altered vascular repair and thrombosis. Modulating the effects of IS using therapeutic strategies targeting oxidative stress and AhR activation could limit the occurrence of cardiovascular disorders in CKD patients.
